# Evidence for differences in DNA methylation between Germans and Japanese

**DOI:** 10.1007/s00414-021-02736-3

**Published:** 2021-11-05

**Authors:** J. Becker, P. Böhme, A. Reckert, S. B. Eickhoff, B. E. Koop, J. Blum, T. Gündüz, M. Takayama, W. Wagner, S. Ritz-Timme

**Affiliations:** 1 Institute of Legal Medicine, University Hospital Düsseldorf, 40225 Düsseldorf, Germany; 2 Institute for Systems Neuroscience, University Hospital Düsseldorf, 40225 Düsseldorf, Germany; 3 Institute of Neuroscience and Medicine, Brain and Behaviour, (INM-7), Research Centre Jülich, 52428 Jülich, Germany; 4 Department of Forensic Medicine, Faculty of Medicine, Fukuoka University, Fukuoka, Japan; 5 Tokyo Medical Examiner’s Office, Tokyo, Japan; 6 Helmholtz Institute for Biomedical Engineering, Stem Cell Biology and Cellular Engineering, RWTH Aachen University Medical School, 52074 Aachen, Germany

**Keywords:** Forensic age estimation, Epigenetic age estimation, DNA methylation, Impact of ancestry/ethnicity

## Abstract

As a contribution to the discussion about the possible effects of ethnicity/ancestry on age estimation based on DNA methylation (DNAm) patterns, we directly compared age-associated DNAm in German and Japanese donors in one laboratory under identical conditions. DNAm was analyzed by pyrosequencing for 22 CpG sites (CpGs) in the genes *PDE4C*, *RPA2*, *ELOVL2*, *DDO*, and *EDARADD* in buccal mucosa samples from German and Japanese donors (*N* = 368 and *N* = 89, respectively).

Twenty of these CpGs revealed a very high correlation with age and were subsequently tested for differences between German and Japanese donors aged between 10 and 65 years (*N* = 287 and *N* = 83, respectively). ANCOVA was performed by testing the Japanese samples against age- and sex-matched German subsamples (*N* = 83 each; extracted 500 times from the German total sample). The median *p* values suggest a strong evidence for significant differences (*p* < 0.05) at least for two CpGs (*EDARADD*, CpG 2, and *PDE4C*, CpG 2) and no differences for 11 CpGs (*p* > 0.3).

Age prediction models based on DNAm data from all 20 CpGs from German training data did not reveal relevant differences between the Japanese test samples and German subsamples. Obviously, the high number of included “robust CpGs” prevented relevant effects of differences in DNAm at two CpGs.

Nevertheless, the presented data demonstrates the need for further research regarding the impact of confounding factors on DNAm in the context of ethnicity/ancestry to ensure a high quality of age estimation. One approach may be the search for “robust” CpG markers—which requires the targeted investigation of different populations, at best by collaborative research with coordinated research strategies.

**Supplementary Information:**

The online version contains supplementary material available at 10.1007/s00414-021-02736-3.

## Introduction

Epigenetic age estimation based on DNA methylation (DNAm) holds the perspective of various forensic applications, e.g., for age estimation in persons without valid identity documents, the identification of unknown deceased, or the identification of the donor of a trace. Many models for the estimation of chronological age based on DNA methylation have been proposed [1–4]; the best of them enabling age estimation with mean absolute errors (MAE) of approximately 3–5 years [5–8].

However, there is a growing perception that DNA methylation may be influenced by exogenous and endogenous factors (for review, see [9–11]) that may be of relevance for age estimation based on DNAm. Against this background, Spolnicka et al. [12] claimed that “studies aiming to identify all potential players influencing differences in DNA methylation at particular loci between individuals at the same chronological age are important […] for better accuracy of age prediction models.”

In this context, the question of population-related differences in DNAm patterns and their impact on forensic age estimation has already been addressed [13–15]. Such differences may be even present at birth [16]. An association between DNAm, histone modifications, and single-nucleotide polymorphisms (SNP) located at specific CpG sites (CpGs) has been interpreted as evidence for the genetic control of DNA methylation [17, 18]. Since SNP allele frequencies may differ considerably among populations of different ancestries, population-related differences in DNAm have been attributed to differences in population-specific alleles or haplotypes [19–21]. Apart from such genetic factors, lifestyle and environmental factors may alter the DNAm pattern. There is strong evidence for such influences from many studies (for review, see [11, 22, 23]). Although the underlying mechanisms are not yet fully understood, there is evidence that genetic variations as well as living conditions (both addressed as “ethnicity/ancestry” in the following) may impact DNAm levels and induce population-related differences [11, 22–24].

Such differences in age-associated DNAm changes between different populations have been described [20, 24–27], and their impact on forensic age estimation based on DNAm has already been discussed [13–15]. Some of these studies were based on the comparison of available Illumina BeadChip datasets of different groups, which may have batch effects that hamper a reliable comparison [24]. In several studies, already developed models were applied by other laboratories to another population, meaning that the samples of the two populations were analyzed in different labs [13, 14]. However, this approach cannot distinguish between methodological and actual population-related differences.

To gain further insight into the relevance of different populations for targeted epigenetic age predictors, DNAm was analyzed by pyrosequencing for 22 CpGs of five genes (*PDE4C*, *RPA2*, *ELOVL2*, *DDO*, and *EDARADD)* in buccal mucosa samples from German and Japanese donors, applying an identical methodological protocol by only one laboratory.

## Material and methods

### Sample collection

Buccal mucosa samples were collected from 368 German donors (203 females, 165 males; ages between 1 month and 94 years) from Germany, mainly from North Rhine-Westphalia and from 89 Japanese donors (55 females, 34 males; ages between 8 and 87 years) after written consent. Twelve of the Japanese donors had been living in Düsseldorf/Germany for several years at the time of sampling. The majority of the remaining 77 Japanese samples were taken in Fukuoka Prefecture (*N* = 59), the rest of the samples came from donors living in Ehime (*N* = 9), Shizuoka (*N* = 5), and Miyazaki (*N* = 3) Prefectures, respectively. For one sample, the exact sampling location was unknown.

### DNA extraction, quantification, and bisulfite conversion

Genomic DNA from buccal swab samples of both groups was extracted using the NucleoSpin® Tissue Kit from Macherey–Nagel (Düren/Germany) according to the manufacturer’s instructions with overnight lysis at 56 °C. DNA was eluted in 100 µl BE buffer (as part of the extraction kit) and DNA extracts were stored at − 20 °C until further analysis. Quantitation was performed following manufacturer’s instructions using either the Applied Biosystems™ 7500 Real-Time PCR System (Waltham, Massachusetts/USA) and the Quantiplex® ProKit (Qiagen, Hilden/Germany) or the QuantiFluor dsDNA Sample Kit (Promega, Madison, Wisconsin/USA) and Quantus Fluorometer (Promega, Madison, Wisconsin/USA).

Bisulfite conversion was performed using either the EZ DNA Methylation-Gold™ Kit (Zymo Research, Irvine, California/USA) or the EpiTect Fast DNA Bisulfite Kit (Qiagen, Hilden/Germany), following the manufacturer’s instructions. If possible, the recommended amount of 200 ng to 500 ng input DNA was used but not less than 10 ng per reaction volume (as recommended in [28]).

### DNA methylation analysis by pyrosequencing (CpGs located in the genes PDE4C, RPA2, ELOVL2, DDO, and EDARADD)

Prior pyrosequencing marker-specific PCRs were performed either using the HotStarTaq Kit (Qiagen, Hilden/Germany) or the PyroMark PCR Kit (Qiagen, Hilden/Germany) under manufacturer’s conditions. Primer sequences were taken from the original papers [29–31]. Hereafter, 10–20 µl of biotinylated PCR product was immobilized to 1 μl Streptavidin Sepharose™HP beads (GE Healthcare, Chicago, Illinois/USA). Sequencing primers were designed as described previously [29–31]. Pyrosequencing was performed using the PyroMark Q24 Advanced CpG Reagents Kit (Qiagen, Hilden/Germany) and the PyroMark Q24 Advanced System (Qiagen, Hilden/Germany).

### Testing for differences between DNAm in German and Japanese samples

In both donor groups, the relationship between DNAm and chronological age was analyzed by linear regression. For all CpGs (located in the genes *PDE4C, RPA2, ELOVL2*, *EDARADD*, and *DDO*), Spearman correlation coefficients (R) were calculated.

Due to the low number of Japanese individuals younger than 10 years and older than 65 years, only German and Japanese individuals with ages between 10 and 65 years (*N* = 287, *N* = 83, respectively) were included in all further analyses.

ANCOVA was performed to detect differences between the DNAm levels in the two populations; at a *p* value < 0.05, the results were considered significant. To further address the effects of different samples sizes and compositions, ANCOVA was performed by testing the Japanese sample (*N* = 83) against age- and sex-matched (all p > 0.3) German subsamples (*N* = 83 each) that were extracted 500 times from the total German group. The medians of these 500 runs were calculated.

### Age estimation based on German training data: modeling

Modeling was based only on the data of individuals with ages between 10 and 65 years and on all CpGs except for *ELOVL2*, CpG 7, and *DDO*, CpG 1 (exhibiting the weakest correlations between DNAm and age with *R* < 0.75).

Age prediction models were trained using a random forest algorithm with (chronological) age as the continuous target variable as well as the 20 CpG information and sex as features for prediction. The prediction forest consisted of 10.000 individual trees that were built from bootstrap samples of the entire dataset using the curvature test. This test selected the split predictor that minimizes the *p* value of chi-square tests of independence between each predictor, i.e., feature, and the response, i.e., age.

Modeling was based on training data consisting of the data of the German donors under the exclusion of extracted German test samples (see below), resulting in a strict separation between training and test data. The performance of the models was tested (a) in the Japanese sample (*N* = 83; Japanese test sample) and (b) in age- and sex-matched (all *p* > 0.3) German subsamples (*N* = 83 each).

### Age estimation based on German training data: performance on the Japanese sample and on age- and sex-matched German test samples

As a measure of prediction accuracy, the mean absolute errors (MAE) were calculated. The performance of age estimation based on the German training data was tested in the Japanese sample (Japanese test sample) and in age- and sex-matched German test samples, respectively. To minimize sampling effects, we did not rely on randomizing the German data into one training and one test set but extracted 83 German test samples 500 times from the total German sample. The 83 German test samples and the 83 Japanese samples were age- and sex-matched, each. Means and medians of the resulting 500 MAEs were calculated for each group.

To detect biases with the consequence of systematic over- or underestimation, the mean deviation of the age gaps (the differences between estimated and chronological ages) was calculated for all 500 runs; the means and medians of the resulting 500 mean deviations were calculated in each group.

## Results

### Buccal swabs from German and Japanese donors: very similar correlation between DNAm levels and age but evidence for significant differences in DNAm at least at two CpG sites

**Fig. 1:**
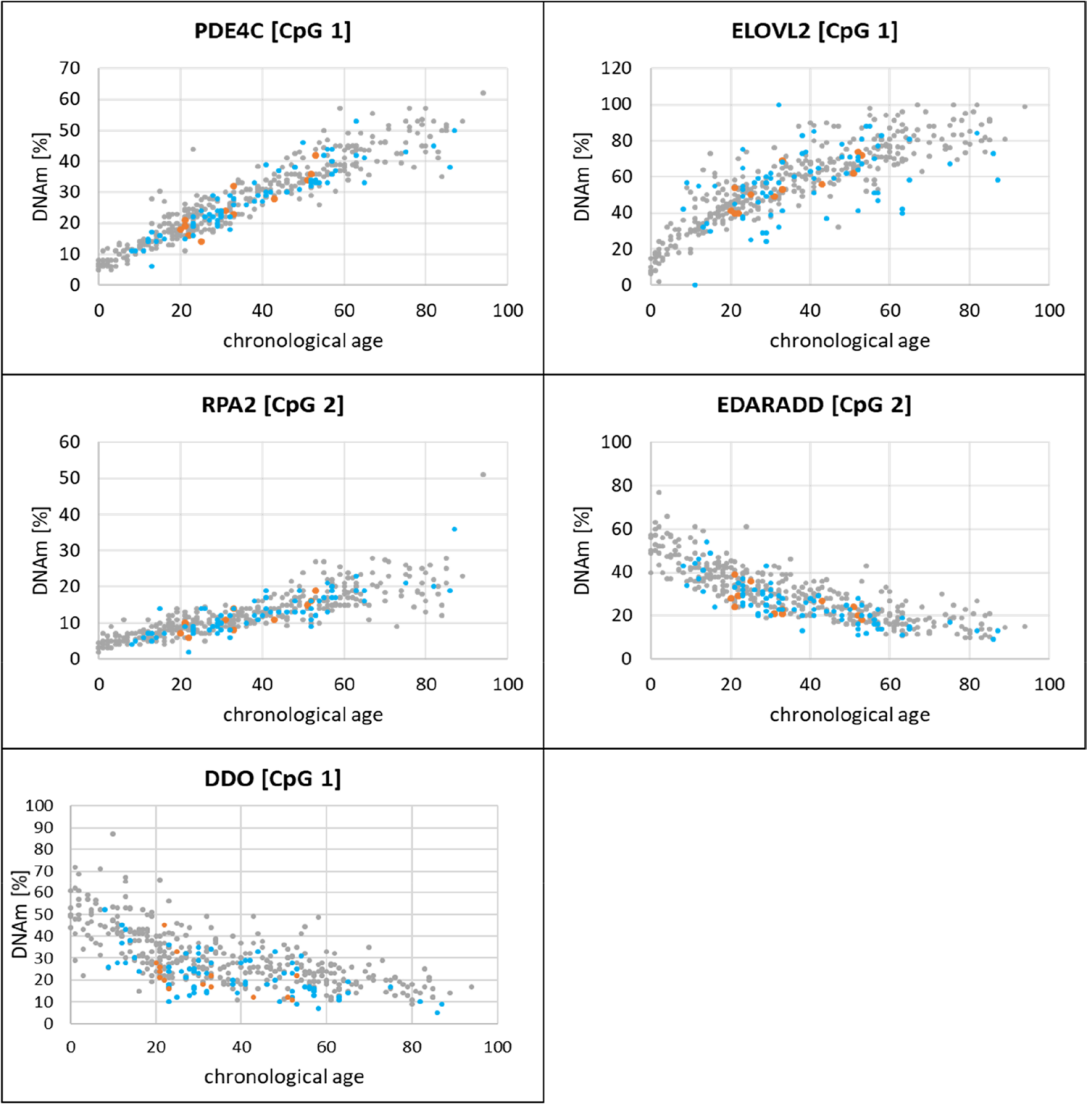
DNA methylation levels (in PDE4C (CpG 1), ELOVL2 (CpG 1), RPA2 (CpG 2), EDARADD (CpG 2), DDO (CpG 1)) in buccal mucosa samples from German (gray, N = 368) and Japanese donors (N = 89, Japanese donors living in Japan = blue (N = 77), Japanese donors living in Germany = orange (N = 12). For genes with more than one analysed CpG site, the data for the CpGs with the highest correlation coefficients (R) are presented. Correlation coefficients (R) for German donors: R(PDE4C, CpG 1) = 0.95, R(ELOVL2, CpG 1) = 0.90, R(RPA2, CpG 2) = 0.89, R(EDARADD, CpG 2) =  − 0.86, R(DDO CpG 1) =—0.73. Correlation coefficients (R) for Japanese donors: R(PDE4C, CpG 1) = 0.93, R(ELOVL2, CpG 1) = 0.89, R(RPA2, CpG 2) = 0.83, R(EDARADD, CpG 2) =  − 0.81, R(DDO CpG 1) =  − 0.62

Analysis of the German and Japanese samples revealed age-associated DNAm levels and a mostly close correlation between DNAm and age in both donor groups, with similar correlation coefficients (Spearman R) between 0.95 (*PDE4C*, CpG1) and 0.67 (*ELOVL2*, CpG 7) in Germans and between 0.93 (*PDE4C*, CpG 1) and—0.62 (*DDO*, CpG 1) in Japanese, respectively (Table 1). In both donor groups, DNAm levels increase at the CpGs of *ELOVL2*, *PDE4C*, and *RPA2* and decrease at *EDARADD* and *DDO* with increasing age (data for DDO and the CpGs of *ELOVL2, PDE4C, EDARADD*, and *RPA2* with the highest correlations between DNAm and age in Fig. 1, for additional data see Supplementary file, Table 3)*.*

**Table 1:** Analyzed CpGs (in the genes PDE4C, ELOVL2, RPA2, EDARADD, and DDO) and Spearman correlation coefficients (R) for the relationship between DNA methylation and age in German and in Japanese samples

				**Spearman correlation coefficient R**	
**Marker**	**Chromosomal location (GRCh38.p13)**	**Position in 450 K array**	**CpG site**	**German (*N* = 287)**	**Japanese (*N* = 89)**
PDE4C [31]	Chr.19: 18,233,106		CpG 1	0.95	0.93
	Chr.19: 18,233,092	cg17861230	CpG 2	0.88	0.81
	Chr.19: 18,233,083		CpG 3	0.82	0.88
	Chr.19: 18,233,080		CpG 4	0.84	0.82
	Chr.19: 18,233,071		CpG 5	0.81	0.86
	Chr.19: 18,233,059		CpG 6	0.81	0.83
	Chr.19: 18,233,049		CpG 7	0.85	0.85
ELOVL2 [29]	Chr.6: 11,044,625		CpG 1	0.90	0.89
	Chr.6: 11,044,629		CpG 2	0.89	0.79
	Chr.6: 11,044,631		CpG 3	0.85	0.83
	Chr.6: 11,044,640		CpG 4	0.86	0.84
	Chr.6: 11,044,642		CpG 5	0.90	0.86
	Chr.6: 11,044,645	cg16867657	CpG 6	0.83	0.85
	Chr.6: 11,044,648		CpG 7	0.67	0.73
	Chr.6: 11,044,664		CpG 8	0.80	0.78
	Chr.6: 11,044,683		CpG 9	0.84	0.79
RPA2 [29]	Chr.1: 27,915,022		CpG 1	0.89	0.81
	Chr.1: 27,915,024		CpG 2	0.89	0.83
	Chr.1: 27,915,067	cg25410668	CpG 3	0.84	0.75
EDARADD [30]	Chr.1: 236,394,371	cg09809672	CpG 1	-0.85	-0.77
	Chr.1: 236,394,383		CpG 2	-0.86	-0.81
DDO [29]	Chr.6: 110,415,571	cg02872426	CpG 1	-0.73	-0.62

CpG 7 in *ELOVL2* and CpG 1 in *DDO* exhibited the weakest correlations between DNAm and age, with R < 0.75. These CpGs were excluded from further analysis.

The DNAm data for the 12 Japanese living in Germany (highlighted in Fig. 1) appear to be very similar to those of equally old Japanese living in Japan. Due to the low number of cases, no further statistical analysis was performed on this question.

Despite the very similar correlation between DNAm and age in Germans and Japanese, there was evidence for significant differences in DNAm at least at two sites. An ANCOVA using the Japanese data and the data of age- and sex-matched German subsamples revealed median *p* values of < 0.05 after 500 runs for *PDE4C* (CpG 2), *RPA2* (CpG3), and *EDARADD* (CpG 2). A median *p* value close to 0.05 (0.0512) was calculated for *ELOVL2* (CpG 8) (Table 2). The significance between the groups was most evident for *EDARADD*, CpG 2 (median *p* = 0.0061, *p* < 0.05 in 88.60% of 500 runs/subsamples) and *PDE4C*, CpG 2 (median *p* = 0.0132, *p* < 0.05 in 79.16% of 500 runs/subsamples). On the other hand side, median *p* values of > 0.3 were calculated for 11 of the analyzed CpGs (Table 2), indicating clearly no differences between Japanese and Germans.

**Table 2:** Results of the statistical testing for differences in DNA methylation (ANCOVA, Japanese sample (N = 83) versus age- and sex-matched German subsamples (N = 83 each)):

Marker	CpG site	Median *p* values	Percentage of runs with *p* < 0.05 (of 500 ANCOVA analyses)
PDE4C	*CpG 1	0.3081	8.74%
	**CpG 2**	**0.0132**	**79.16%**
	CpG 3	0.1464	23.78%
	*CpG 4	0.5938	0.94%
	*CpG 5	0.6283	0.68%
	*CpG 6	0.4850	2.52%
	*CpG 7	0.6040	1.36%
ELOVL2	*CpG 1	0.5537	1.74%
	*CpG 2	0.5281	1.68%
	*CpG 3	0.3686	7.60%
	CpG 4	0.2643	12.02%
	*CpG 5	0.4390	2.84%
	CpG 6	0.1814	19.30%
	CpG 8	0.0512	49.20%
	CpG 9	0.0932	33.60%
RPA2	*CpG 1	0.4528	1.50%
	*CpG 2	0.5426	1.98%
	CpG 3	0.0391	56.88%
EDARADD	CpG 1	0.0661	41.72%
	**CpG 2**	**0.0061**	**88.60%**

Strong evidence for significant differences for PDE4C, CpG2 and EDARADD, CpG 2 (highlighted in gray and bold, median p < 0.05, high percentages of analyses with p < 0.05), some evidence for significant differences also for RPA2, CpG3 (highlighted in grey, median p < 0.05, in more than 50% of the runs with p < 0.05), no evidence for differences (p > 0.3, marked with an asterisk)

### Age prediction by models based on German training data did not reveal relevant differences between the Japanese sample and the age-and sex-matched German test samples

**Fig. 2:**
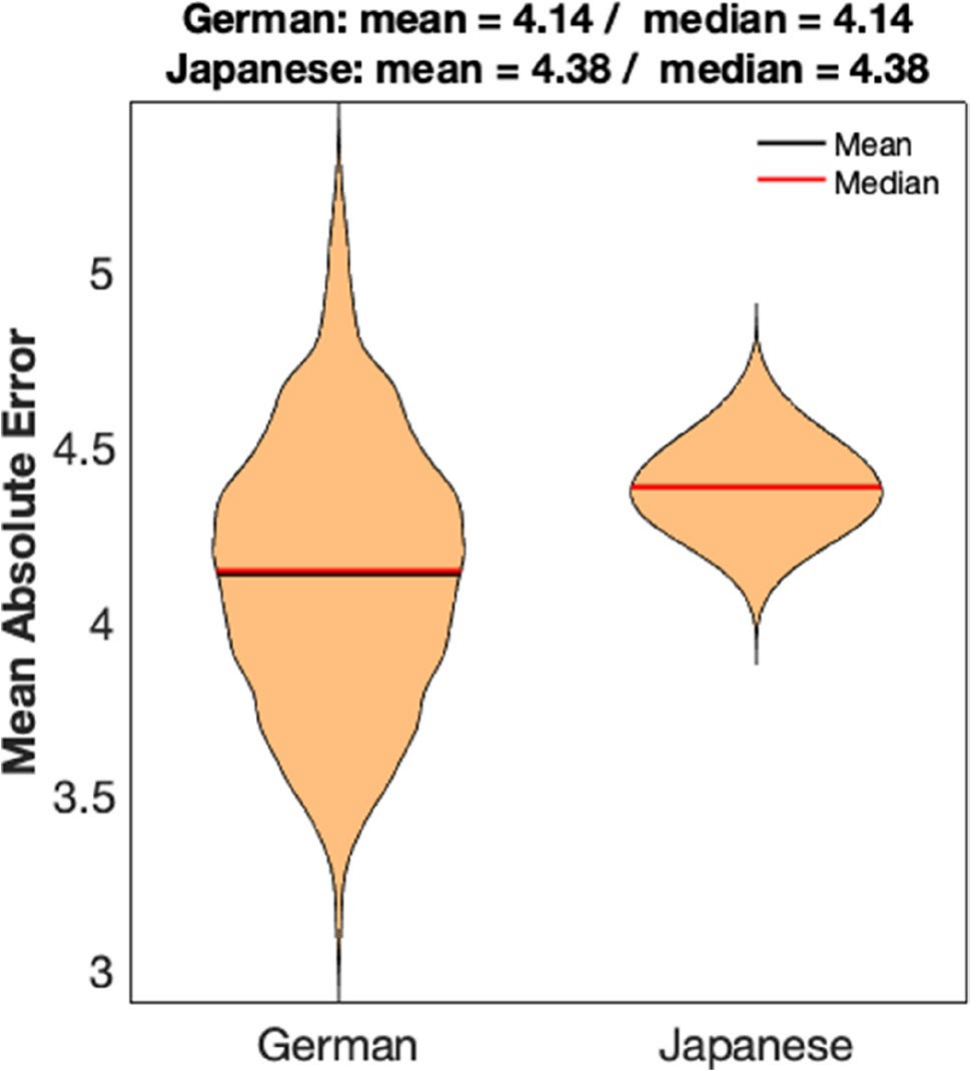
Mean absolute errors (MAE, in years) of age estimation based on the German trainings data (“German” = German test subsamples (N = 83 each), “Japanese” = Japanese sample (N = 83)). Modeling was based on the German training data under exclusion of 500 extracted age- and sex-matched German test subsamples, respectively. The figure depicts the MAEs for 500 analyses for each group; the greater scattering of MAEs in the Germans is due to the extraction of 500 different German test groups, whereas the Japanese test group is the same group in all 500 analyses

The means and medians of the MAEs calculated in 500 runs for the Japanese sample (Japanese test sample) and 500 different extracted age- and sex-matched German test samples were very similar (Germans: 4.14 years (mean), 4.14 years (median); Japanese: 4.38 years (mean), 4.38 years (median); Fig. 2).

**Fig. 3:**
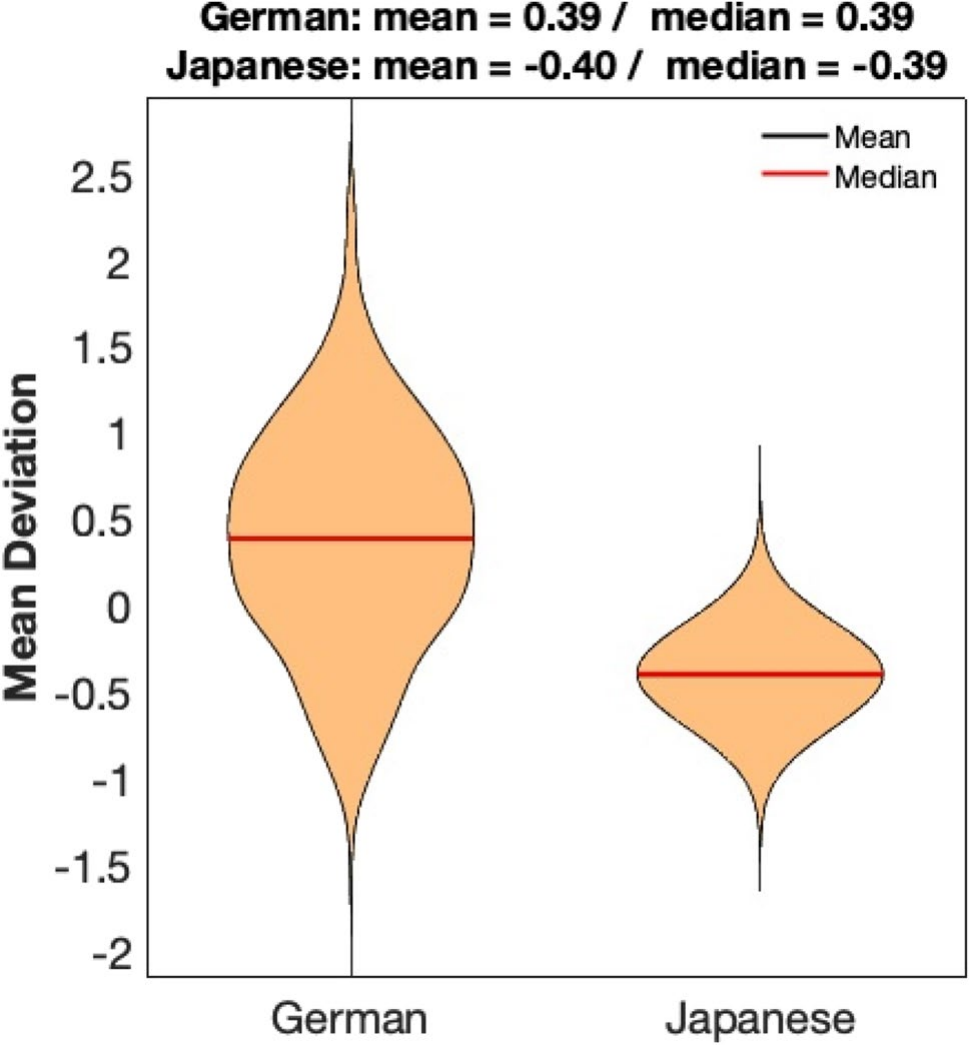
Mean deviation of the age gaps (difference between estimated and chronological ages, in years) after age estimation of the Japanese group (N = 83) and German test subsamples (N = 83 each) based on the German trainings data. Modeling was based on the German training data under exclusion of 500 extracted age- and sex-matched German subsamples, respectively. The figure depicts the mean deviations after 500 analyses for each group; the greater scattering of the German data is due to the extraction of 500 different German test groups, whereas the Japanese test group is the same group in all 500 analyses

There was no clear indication for biases resulting in a relevant systematic over- or underestimation of age, since the means as well as the medians of the mean deviation of the age gaps (from 500 runs) were very low in both test groups (Germans: 0.39 years (mean), 0.39 years (median); Japanese: − 0.40 years (mean), − 0.39 years (median); Fig. 3).

## Discussion

The primary aim of this study was to contribute to the discussion about the possible effects of ethnicity/ancestry on age estimation based on DNAm by a direct comparison of DNAm patterns in German and Japanese samples that were analyzed in one lab under identical conditions. However, due to the interindividual variability of DNAm within a population [32–34], very large numbers of samples have to be analyzed to reliably prove differences between populations. Thus, the numbers of samples analyzed here (287 German samples, 83 Japanese samples in the age range 10–65 years) is a clear limitation of this study. Moreover, the size and the composition of the two groups were very different.

To overcome these limitations at least partly, we did not compare the Japanese group with only one age- and sex-matched German group, but with 500 age- and sex-matched German subgroups that were extracted from the total German group. The median *p* values of 500 ANCOVA analyses allow much more robust conclusions than the *p* values derived from just one analysis that includes only one randomly extracted German subgroup.

The strategy of subsampling was also applied in testing the performance of age prediction (based on the German training data set) in German test groups versus the Japanese test group. In each run, the model for age estimation was calculated only on the basis of the remaining training data, thus allowing a strict separation between training data and test data. That means that age estimation was performed in the Japanese test sample and in age- and sex-matched German test samples by 500 different models. The median MAEs give a robust impression of the performance of age estimation in the German and Japanese group. This strategy is an approach to reduce the impact of sampling effects, if the number of samples is limited.

The methodological approach of subsampling may be unusual and does not allow to present one model for age estimation (since, in fact 500 models were used). However, the aim of our work was not the presentation of a new model but to gain further insight into the question of the relevance of ethnicity/ancestry for age estimation based on DNAm.

In both German and Japanese samples, the DNA-methylation levels in buccal swabs were age-associated at all analyzed CpGs (in *PDE4C, RPA2, ELOVL2*, *EDARADD*, and *DDO*). This finding was to be expected, since similar data have already been published [5, 13, 29–31]. Differences in correlation coefficients (Table 1) were only small and may not be overinterpreted in light of the different (and in the Japanese group) limited number of samples in the donor groups.

Although the correlation between DNAm and age was very similar in Germans and Japanese, there was evidence of differences between the two groups in DNAm at some CpGs sites, most noticeable in *EDARADD* (CpG 2) and *PDE4C* (CpG 2). For these sites, the median *p* values of the 500 ANOVAs were *p* = 0.0061 (*EDARADD* (CpG 2)) and 0.0132 *(PDE4C* (CpG 2)); *p* values < 0.05 were calculated in 88.60% and 79.16% of the 500 runs, respectively (Table 2). These results can be interpreted as strong evidence for significant differences of DNAm at *EDARADD* (CpG 2) and *PDE4C* (CpG 2)*.* The ANCOVA results for CpG 3 in *RPA2* (median *p* value of 0.0391, *p* values < 0.05 in 56.88% of the 500 runs) at least suggest differences between the two groups.

One can only speculate about the biological background of such differences in the DNA methylation pattern between Germans and Japanese. Basically, genetic variations as well as living conditions may play a role [11, 22, 23]. The Japanese population may be genetically more homogeneous than other populations [35–37]. If so, also genetically determined DNA methylation patterns may be more homogeneous in the Japanese population, making differences to other populations more prominent. The finding that the DNAm data for the Japanese living in Germany appeared to be within the range of the other Japanese data may be another indication for the relevance of genetic factors; however, only 15 Japanese living in Germany were examined. It would be interesting to conduct further research on this topic under the inclusion of a higher number of cases.

If there is evidence for differences in the DNA methylation pattern at some CpGs, the question arises, if this may be relevant for age estimation models that include DNAm data of such CpGs. Our results demonstrated that means and medians of the MAEs were very similar in the German and Japanese cohort, and there was no clear indication for biases resulting in a relevant systematic over- or underestimation of age.

These findings do not contradict the evidence for significant differences in the DNAm levels at some CpGs in different genes. The age prediction models were based on data of 20 CpGs, for only two (to three) of them the ANCOVA analyses revealed evidence for differences between Japanese and Germans. Obviously, the high number of included “robust CpGs” (11 CpGs exhibited median *p* values > 0.3, see Table 2) prevented relevant effects of the differences in DNAm at two CpGs.

Nevertheless, our findings emphasize the impact of the ethnicity/ancestry on DNAm and are in line with the findings of other groups. Cho et al. [14] applied the age prediction model of Zbieć-Piekarska et al. [38] (derived from a Polish population, markers located in the genes *ELOVL2, C1orf132, TRIM59, KLF14*, and *FHL2* genes) to blood samples from 100 Koreans. The authors reported that the age predictive performance of the model “is relatively consistent across different population groups,” although “the extent of the age association in Koreans was not identical to that of the Polish,” in particular at the loci *FHL2* and *C1orf132*. Fleckhaus et al. [15] analyzed DNAm at five CpG sites in the genes *ASPA*, *ITGA2B*, *PDE4C*, and *ELOVL2* in buccal mucosa samples of three independent population groups from the Middle East, West Africa, and Central Europe and reported “first evidence that the strength of correlation between methylation and chronological age and thus the accuracy of age prediction may vary between populations.” Thong et al. [13] analyzed blood samples from a local population comprising Chinese, Malays, and Indians (CpG sites in the genes *ELOVL2, KLF14, TRIM59*, and *FHL2)* and established age prediction models on the basis of the data from all three subpopulations. Using this model, they did not observe significant age prediction errors among the Chinese, Malays, and Indians. In contrast, notable differences in prediction accuracy were observed when the model was applied to a Polish and a French population (by using DNAm data reported by [5, 38]), the Polish samples were systematically underestimated. As possible reasons for these differences, the authors propose “methodology and instrumental variations during bisulfite conversion and/or pyrosequencing.”

Such methodological effects can be excluded for the here presented data of buccal mucosa samples of German and Japanese donors. These data suggest significant differences between the investigated populations in the methylation of at least two analyzed CpGs (*EDARADD* (CpG 2) and *PDE4C* (CpG 2)). Based on the presented data it cannot deduced, if the findings are just a matter of these two populations (Germans/Japanese), the very possibility of such a problem should lead to caution.

Forensic science should further address the influence of ethnicity/ancestry to optimize the potential of age estimation based on DNAm; a need for research has been already stated by others [9, 13, 15, 39, 40]. Thong et al. [13] suggested the retraining of age prediction models, if they are to be applied to individuals of other populations. This suggestion implies that retrained models are developed for all relevant populations and that the assignment of an unknown donor of a trace or a non-identified deceased to a specific population is known. Fleckhaus et al. [15] proposed “to include ancestry informative markers into the analysis as an additional factor for age prediction models.” Another approach would be to identify “robust” CpGs as basis for age prediction models that can be used regardless of the population of origin. Whatever approach is chosen, the targeted investigations of different populations are required at best by collaborative research with coordinated research strategies.

## Supplementary Information

Below is the link to the electronic supplementary material.

Supplementary file1 (XLSX 80 KB)
